# Simvastatin targets the IL-6 signalling cascade in human melanoma cells

**DOI:** 10.1186/1471-2210-11-S2-A25

**Published:** 2011-09-05

**Authors:** Christine Wasinger, Christoph Minichsdorfer, Martin Hohenegger

**Affiliations:** 1 Institute of Pharmacology, Center of Physiology and Pharmacology, Medical University of Vienna, 1090 Vienna, Austria

## Background

In the last years the number of melanoma patients has increased markedly. High plasma levels of interleukin-6 (IL-6) are associated with bad prognosis and reduction in overall survival of these patients. Statins, HMG-CoA reductase inhibitors, are well-tolerated therapeutics for hypercholesterolemia. We have recently shown that simvastatin triggers apoptosis in 518A2 human melanoma cells which is paralleled by concentration-dependent changes in autocrine IL-6 secretion. Here, we investigated the impact of simvastatin on the IL-6 pathway.

## Methods

We investigated the expression and distribution of the heteromeric IL-6 receptor (IL-6-R/gp130) and its down-stream pathway in 518A2 human melanoma cells by FACS, Western blot and real-time PCR analysis. Furthermore, we chose a fluorescent fusion protein (STAT3-YFP) plasmid construct to study the influence of simvastatin on signalling down-stream of the IL-6 receptor.

## Results

Increasing concentrations of simvastatin led to enhanced surface expression of the IL-6-R and the gp130 subunit. Cells with higher IL-6 holo-receptor expression detached, but were still viable. In Western blot analysis the precursor level of the less glycosylated gp130 subunit was enriched in simvastatin samples, which was hand in hand with enrichment of mature gp130 receptor. The mRNA levels of IL-6-R and gp130 were not regulated by the statin treatment. Stimulation with IL-6 of the STAT3-YFP-tranfected 518A2 cells resulted in the formation of cytosolic density spots of STAT3-YFP, which co-localized with lysosomal markers. Simvastatin significantly delayed the accumulation of STAT3 in these dots. The suppressor of cytokine signalling (SOCS) proteins (SOCS1, SOCS3), which may play a crucial role in the inhibition of the IL-6 pathway, were not regulated by simvastatin on protein or mRNA level.

## Conclusions

These data indicate that statins like simvastatin are capable of interfering with the IL-6 pathway on the heteromeric IL-6 receptor, as expression is increased on the cell surface. Furthermore, simvastatin affected the dot formation of STAT3, which occurred significantly later compared to the untreated cells. However, simvastatin treatment is not able to impair the level of the negative feed-back protein SOCS3.

